# Loss, Gain, and Retention: Mechanisms Driving Late Prophase I Chromosome Remodeling for Accurate Meiotic Chromosome Segregation

**DOI:** 10.3390/genes13030546

**Published:** 2022-03-19

**Authors:** Laura I. Láscarez-Lagunas, Marina Martinez-Garcia, Monica P. Colaiácovo

**Affiliations:** 1 Department of Genetics, Harvard Medical School, Boston, MA 02115, USA; laura_lascarezlagunas@hms.harvard.edu; 2 Department of Biotechnology-Plant Biology, School of Agricultural, Food and Biosystems Engineering, Universidad Politécnica de Madrid, 28040 Madrid, Spain; marina.martinezg@upm.es

**Keywords:** meiosis, bivalent, chromosome remodeling, SC disassembly, chromosome segregation, sister chromatid cohesion, crossover recombination

## Abstract

To generate gametes, sexually reproducing organisms need to achieve a reduction in ploidy, via meiosis. Several mechanisms are set in place to ensure proper reductional chromosome segregation at the first meiotic division (MI), including chromosome remodeling during late prophase I. Chromosome remodeling after crossover formation involves changes in chromosome condensation and restructuring, resulting in a compact bivalent, with sister kinetochores oriented to opposite poles, whose structure is crucial for localized loss of cohesion and accurate chromosome segregation. Here, we review the general processes involved in late prophase I chromosome remodeling, their regulation, and the strategies devised by different organisms to produce bivalents with configurations that promote accurate segregation.

## 1. Chromosome Remodeling

Sexual reproduction relies on the production of haploid gametes (i.e., eggs and sperm) from diploid germ cells during meiosis. The meiotic cell division program reduces the diploid chromosome number by half, by following a single round of DNA replication, with two rounds of cell division. In the first meiotic division (MI), homologous chromosomes segregate away from each other; whereas, in the second meiotic division (MII), sister chromatids separate [1]. During meiotic prophase I, homologous chromosomes organize into pairs, connected by crossovers and sister chromatid cohesion (bivalents) [2]. Bivalents are restructured during meiotic prophase I, to achieve a configuration that promotes their proper attachment to opposite poles of the MI spindle, and subsequent accurate meiotic chromosome segregation; therefore, errors in bivalent structural remodeling result in improper chromosome segregation, embryonic lethality, and birth defects [3].

Chromosome remodeling is a key feature of meiosis. During early prophase, events such as the formation of programmed DNA double-strand breaks (DSBs), repair of a subset of these DSBs as interhomolog crossover recombination events, and assembly of the zipper-like scaffold, known as the synaptonemal complex (SC), between homologs require both localized as well as global changes in the chromosome structure. During late prophase, the structural remodeling of chromosomes involves changes in chromosome condensation, disassembly of the SC, and restructuring around the crossover site. Here, we will focus on the mechanisms underlying proper chromosome remodeling during late prophase I (pachytene to diakinesis transition), because of their importance in achieving a configuration, observed by late diakinesis/pro-metaphase I for bivalents, that promotes bipolar spindle attachment and localized loss of cohesion, summarizing the current knowledge for different organisms.

## 2. Crossover Formation Triggers Chromosome Remodeling in Late Prophase I

Meiotic crossovers (COs) are formed via the homology-directed repair of programmed DSBs, and both their frequency and distribution are tightly regulated. For example, every pair of homologous chromosomes must undergo at least one CO (obligate crossover), and when two or more COs are formed along a chromosome, they do not tend to occur near one another (crossover interference) [4,5]. Although there are exceptions (reviewed in [6]), COs are essential for accurate chromosome segregation in most organisms. Specifically, COs in conjunction with sister chromatid cohesion (SCC) result in structures referred to as “chiasmata”, which tether homolog pairs, thereby facilitating their correct orientation on the MI spindle [7,8,9].

In most organisms, for CO formation, chromosomes are required to locate and pair with their homologs, and stabilize this association via assembly of the synaptonemal complex (SC) [2]. The SC is a proteinaceous structure whose organization is widely conserved. As revealed by electron microscopy, it consists of two lateral elements, formed by axis-associated proteins along each homolog, that flank a central region with proteins bridging both axes, resulting in a zipper-like structure [2,10,11]. The SC is required to stabilize the interactions between pairs of homologous chromosomes until COs are formed, as shown in budding yeast, worms, flies, and mice [12,13,14,15,16,17,18,19,20], and to prevent the formation of closely spaced COs in *Arabidopsis* [21,22].

**Figure 1:**
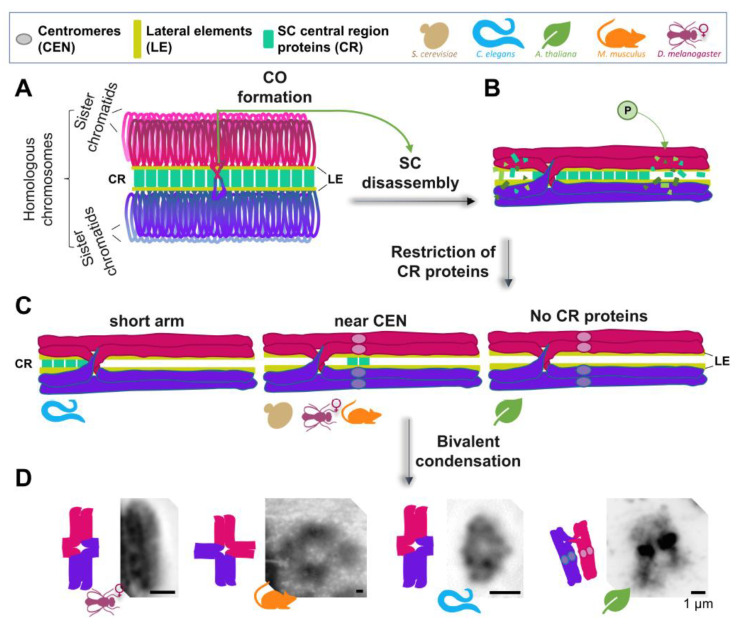
Meiotic bivalent remodeling in different model organisms. (**A**) A pair of fully synapsed homologous chromosomes (in magenta and purple) is shown, with only the lateral element (LE) and central region (CR) proteins of the synaptonemal complex (SC) being highlighted for simplicity. (**B**) Upon interhomolog crossover (CO) formation, different types of regulation may trigger SC disassembly, starting in late pachytene (shown is the phosphorylation of SC proteins). (**C**) SC disassembly can result in either retention/restriction of the CR proteins of the SC to a particular subregion of the bivalents (such as the “short arm” in *Caenorhabditis elegans*, or near the centromeres in several other organisms), or the complete removal of CR proteins, such as in *Arabidopsis thaliana*. The process of late prophase I bivalent remodeling includes changes in chromatin condensation and compaction, to produce the final bivalent structure, characteristic of diakinesis/prometaphase I. (**D**) Representative images of DAPI-stained bivalents at this stage are shown for *Drosophila melanogaster* (courtesy of Stacie Hughes and Scott Hawley, Stowers Institute for Medical Research), *Mus musculus* (courtesy of Tegan S. Horan and Paula Cohen, Cornell University), *C. elegans*, and *A. thaliana* (courtesy of Monica Pradillo, Universidad Complutense de Madrid), to provide examples of the conservation and diversity of bivalent morphology.

CO formation, in turn, triggers chromosome remodeling in late prophase I, which involves the coordinated loss, gain, retention and/or relocalization of several important proteins along different chromosome subdomains. The end result of this chromosome remodeling is a configuration that will ensure the regulated removal of SCC, bipolar attachment at the spindle, and subsequent accurate chromosome segregation at MI (Figure 1). After CO formation, the SC begins to disassemble in an asymmetric manner, and SC proteins are either retained at specific chromosome subdomains until late prophase I (worms, budding yeast, flies, and mice) or completely removed from the chromosomes (plants).

In *C. elegans*, which carry holocentric chromosomes (centromere activity is distributed throughout the chromosome length) and exhibit absolute CO interference, a single CO is formed per homologous chromosome pair [23]. Furthermore, since this CO usually occurs in an off-center position along the length of the chromosome, late prophase remodeling results in six cruciform-shaped asymmetric bivalents, with distinct long and short arms [24,25,26]. Following CO formation, chromosome remodeling is initiated with the asymmetric disassembly of the SC. Central region proteins (SYP-1/2/3/4/5/6) are lost along the longest distance from the site of the CO to a chromosome end (the long arm of the bivalent), and persist on the short arms of the bivalent [27,28,29]. In contrast, the HORMA domain, containing the axis-associated proteins HTP-1 and HTP-2, is lost from the short arms of the bivalent and retained on the long arms [30]. The axis-associated components HTP-3 and HIM-3 retain a relatively uniform distribution along the length of the chromosome axes, and remain associated with both the long and short arms of the bivalent [27,31]. In late diakinesis, the disassembly of the SC proteins on the short arm requires the conserved nuclear protein Akirin (AKIR-1 in worms), which acts through a mechanism that is still unknown [32]. The asymmetric disassembly of the SC central region components, dictated by the position of the CO, was proposed to play an important role in regulating the loss of SCC at MI in *C. elegans* [27]. This was directly tested through DSB induction at specific genomic positions, by *Mos1* transposon excision in *spo-11* mutants lacking endogenous meiotic DSB formation, which revealed that the CO position in early meiotic prophase must be tightly regulated for proper designation of chromosome subdomains and chromosome remodeling, resulting in the regulated loss of sister chromatid cohesion and accurate chromosome segregation in *C. elegans* [33].

In organisms with monocentric chromosomes (with a discrete or confined centromere), such as yeast and flies, the SC also disassembles asymmetrically, and persists at centromeres until late prophase [34,35,36]. Similarly, in mouse spermatocytes, the SC is retained at paired centromeres, after it is lost along the length of the chromosomes [37,38]. In budding yeast, as the SC disassembles, the central region component Zip1 is preferentially retained at the centromeres, where it remains associated until the centromeres are attached to microtubules. Furthermore, evidence suggests that this localized retention promotes centromere biorientation, to ensure homologous chromosome segregation to opposite poles at meiosis I [34]. In flies, the SC starts to progressively disassemble along the chromosome arms during mid prophase I, and is completely disassembled from the arms by late prophase I. This loss of the SC coincides temporally with the loading of condensin complex components [39]. Interestingly, SC components, such as the central region proteins C(3)G and Cona, as well as the cohesin subunit SMC1, remain associated with the centromeres beyond euchromatic SC disassembly [36]. In mouse spermatocytes, the SC proteins that persist at the chiasmata, after SC disassembly, are proposed to regulate local remodeling of homologous chromosome axes, thereby promoting centromere and chiasma functions. In mice, instead of triggering global chromosome remodeling, COs are proposed to generate local changes in SCC and chromatin condensation [37]. CO-triggered local changes/destabilization of chromosome axes have been described in other organisms. In rat spermatocytes at diplotene, the bridges between homologs, observed at chiasma sites, are not stained by antibodies that recognize the meiosis-specific cohesin REC8; however, REC8 is present on the axes flanking the chiasma [40]. Additionally, in *Sordaria*, it has been demonstrated, by EM, that sister chromatid axes are found to be locally separated at CO sites [41]. Plants, such as *A. thaliana*, undergo a complete loss of the SC central region proteins from the bivalents during late prophase I remodeling [42], while the ASY1 lateral element protein can persist [43], occasionally in strange “tinsel-like” diplotene structures, such as in wheat and barley [44].

## 3. Restructuring of the Bivalent to Form a Compact Structure

### 3.1. Regulation of SC Disassembly

Cell cycle kinases and post-translational regulation of SC components play an important role in regulating SC disassembly (reviewed in [45,46]). Yeast requires Cdc5 (Polo-like kinase), Ipl1 (Aurora B), DDK (Dbf4-dependent Cdc7 kinase), and CDK1 for SC disassembly [47,48,49]. However, their targets that lead to SC disassembly remain unclear. PLK1 and Aurora B are also required for SC disassembly in mice. In this case, PLK1 directly phosphorylates the SC central element proteins SYCP1, TEX12, and SYCE1 during meiotic prophase, helping with the disassembly of the central region and lateral element proteins of the SC [50]. Aurora B and the inner centromere protein (INCEP) also localize to the meiotic chromosomes during prophase [51]. However, unlike PLK1, Aurora kinase activity is required exclusively for disassembly of the lateral elements [52]. CDK1–cyclin B is also involved in this process [53,54,55]. Currently, there is no evidence suggesting that cell cycle kinases are involved in SC disassembly in flies. However, many of these kinases are present during late prophase I female meiosis in flies, so it is possible that their role in disassembly is yet to be uncovered. The involvement of mitogen-activated protein (MAP) kinase has been revealed recently in *C. elegans*. Specifically, ECT-2, the mammalian Rho guanine nucleotide exchange factor homolog, working through the conserved RAS/ERK MAP kinase signaling pathway, regulates the disassembly of the SC, via direct phosphorylation of the central region component SYP-2 [56]. Additionally, regulation of the HIM-3 phosphorylation status is required for timely disassembly of SC central region proteins from the long arm, and for proper timing of HTP-1 and HTP-2 dissociation from the short arm [57].

### 3.2. Changes in Chromosome Condensation and Compaction

The late prophase I chromosome remodeling process involves changes in chromosome condensation and compaction, by which meiotic chromosomes are transformed from the elongated linear structures visible at mid-pachytene into the condensed, highly asymmetrical cross-shaped bivalent structures present at diakinesis (worms and mice) and prometaphase I (flies), or into “rings” or “rods” (plants). The multi-subunit condensin complex is responsible for chromosome condensation in multiple organisms. At least two different types of condensin complexes, known as condensin I and II, exist among eukaryotes [58]. The two complexes share the same pair of SMC2 and SMC4 subunits, both belonging to the structural maintenance of chromosomes (SMC) family of chromosomal ATPases. Additionally, each complex has a unique set of three non-SMC subunits (i.e., CAP-D2, CAP-G, and CAP-H for condensin I, and CAPD3, CAP-G2, and CAP-H2 for condensin II) (reviewed in [59]).

In *Saccharomyces cerevisiae*, the single condensin complex localizes to the axial core of pachytene chromosomes and contributes to their axial compaction [60]. Chromosome bridges are often observed during anaphase I in condensin mutants, indicating that condensin function is required for proper chromosome segregation. Condensin has also been shown to help recruit Polo/Cdc5 to meiotic chromosomes, which, in turn, contributes to cohesin release, through its direct phosphorylation [61]. A four-subunit complex, known as monopolin, plays a central role in co-orienting sister kinetochores during meiosis I [62,63]. Condensin contributes to properly localizing a monopolin subunit (Mam1) at kinetochores, thereby ensuring co-orientation [64].

Studies in *A. thaliana* suggest that condensin I might be sufficient to support meiotic chromosome condensation, and, therefore, proper chromosome segregation, in plants [65,66,67]. Among the three condensin complexes present in *C. elegans*, only condensin II associates with sister chromatids during diplotene and diakinesis, and mediates their compaction and resolution, contributing to the formation of compact and well-resolved cruciform bivalents [31]. Additionally, AKIR-1 affects the condensation of the bivalents, independently of condensin [32]. In *Drosophila*, CAP-G, a condensin I subunit, is required for SC disassembly, and to keep chromosomes in their metaphase I configuration in female meiosis [39]. Based on the results from antibody injection experiments, it has been proposed that condensins I and II play distinct roles during the construction of bivalent chromosomes in mouse oocytes; condensin II is involved in resolving sister chromatid axes, whereas condensin I may assemble or reinforce a unique centromeric structure, contributing to the monopolar attachment of sister kinetochores [68]. In addition to condensin complexes, other axis-associated components, such as topoisomerase II, also seem to participate in the compaction of bivalents during late prophase I remodeling in several organisms [69,70,71].

### 3.3. Regulation of Loss of Cohesion

The loss, gain, and retention of proteins in the newly established subdomains, generated during late prophase I chromosome remodeling, is critical for the regulated stepwise loss of SCC [25,27,30,72]. In monocentric organisms, such as yeast, flies, *Arabidopsis*, and mice, both the SC and cohesin are lost along the chromosome arms, but persist at the centromere to ensure that sister chromatids segregate together, probably by promoting proper centromere biorientation (reviewed in [73]). Once homolog biorientation has occurred, separase becomes active. Separase cleaves the meiosis-specific cohesin Rec8 at the chromosome arms only, while, in centromeric regions, Rec8 is protected from separase activity by the presence of shugoshin. Rec8 phosphorylation is essential for its cleavage by separase [74,75,76,77,78]. At the pericentromere, members of the shugoshin/Mei-S332 protein family counteract this phosphorylation by recruiting a specific form of the protein phosphatase 2A [79,80,81], retaining cohesion in this region until meiosis II. In *C. elegans*, which has holocentric chromosomes, upon loss of the residual SC central region proteins, localized at the short arms of the bivalent at late diakinesis, the Aurora B kinase homolog AIR-2 localizes to the short arm, where it phosphorylates REC-8, allowing its cleavage by separase at anaphase I, and subsequent segregation of homologous chromosomes to opposite spindle poles [27,82]. LAB-1, the functional analog of shugoshin, which, during earlier prophase, was localized throughout the length of the chromosomes, is restricted to the long arms by late prophase I, protecting SCC along this domain from AIR-2 phosphorylation and premature removal, by targeting GSP-1 and GSP-2 (PPI protein phosphatase homologs) to this chromosomal region [72,83,84]. In addition, the chromatin-associated protein HIM-17 has been implicated in preventing the loading of AIR-2 on the long arm of the bivalent, by regulating the expression, localization, and possibly the phosphorylation of GSP-1 and GSP-2 [85].

## 4. Concluding Remarks/Open Questions

Here, we summarized the progress made in elucidating the mechanisms regulating chromosome remodeling during late prophase I. Although different organisms use distinct strategies, late prophase I chromosome remodeling is a conserved and crucial process that ensures the regulated loss of sister chromatid cohesion, bipolar attachment of homologs at the metaphase I spindle, and accurate chromosome segregation, avoiding aneuploidy. One of the main differences among species derives from whether their chromosomes are monocentric or holocentric. In monocentric organisms, the centromere serves as the focal point for mechanisms that serve to shape the remodeling process, such as co-orienting sister chromatids and promoting the local protection of cohesion to allow for the two-step release of SCC. Holocentric organisms apply strategies, such as using the CO position as the symmetry breaking point that subdivides the bivalent into distinct subdomains with different fates, that can promote the localized removal of SCC.

Although some of the basic mechanisms underlying late prophase I chromosome remodeling are understood, there are still many unanswered important questions, including the following: What kind of signals are used to indicate CO formation and trigger post-translational modifications of SC proteins and asymmetric SC disassembly? What mechanisms assess the distance between a CO and the ends of the chromosomes, to determine subdomains in holocentric chromosomes? What other proteins/mechanisms of regulation are implicated in asymmetric SC disassembly? How is SC disassembly connected with changes in chromosome condensation? Further studies in different organisms will reveal additional levels of regulation and identify new proteins, contributing to a better understanding of chromosome remodeling in late prophase I.
